# Intervention to prevent further falls in older people who call an ambulance as a result of a fall: a protocol for the iPREFER randomised controlled trial

**DOI:** 10.1186/1472-6963-13-360

**Published:** 2013-09-27

**Authors:** A Stefanie Mikolaizak, Paul M Simpson, Anne Tiedemann, Stephen R Lord, Gideon A Caplan, Jason C Bendall, Kirsten Howard, Jacqueline CT Close

**Affiliations:** 1 Neuroscience Research Australia, University of New South Wales, Barker Street, Randwick, 2031 Sydney, NSW, Australia; 2 Ambulance Service of New South Wales, Sydney, Australia; 3 The George Institute for Global Health, The University of Sydney, Sydney, Australia; 4 Prince of Wales Clinical School, University of New South Wales, Sydney, Australia; 5 School of Public Health, University of Sydney, Sydney, Australia

**Keywords:** Ambulance, Accidental falls, Aged, Prevention, Intervention

## Abstract

**Background:**

An increasing number of falls result in an emergency call and the subsequent dispatch of paramedics. In the absence of physical injury, abnormal physiological parameters or change in usual functional status, it could be argued that routine conveyance by ambulance to the Emergency Department (ED) is not the most effective or efficient use of resources. Further, it is likely that non-conveyed older fallers have the potential to benefit from timely access to fall risk assessment and intervention. The aim of this randomised controlled trial is to evaluate the effect of a timely and tailored falls assessment and management intervention on the number of subsequent falls and fall-related injuries for non-conveyed older fallers.

**Methods:**

Community dwelling people aged 65 years or older who are not conveyed to the ED following a fall will be eligible to be visited at home by a research physiotherapist. Consenting participants will receive individualised intervention strategies based on risk factors identified at baseline. All pre-test measures will be assessed prior to randomisation. Post-test measures will be undertaken by a researcher blinded to group allocation 6 months post-baseline. Participants in the intervention group will receive individualised pro-active fall prevention strategies from the clinical researcher to ensure that risk factors are addressed adequately and interventions carried out. The primary outcome measure will be the number of falls recorded by a falls diary over a 12 month period. Secondary outcome measures assessed six months after baseline will include the subsequent use of medical and emergency services and uptake of recommendations. Data will be analysed using the intention-to-treat principle.

**Discussion:**

As there is currently little evidence regarding the effectiveness or feasibility of alternate models of care following ambulance non-conveyance of older fallers, there is a need to explore assessment and intervention programs to help reduce subsequent falls, related injuries and subsequent use of health care services. By linking existing services rather than setting up new services, this pragmatic trial aims to utilise the health care system in an efficient and timely manner.

**Trial registration:**

Australian New Zealand Clinical Trials Registry: ACTRN 12611000503921

## Background

More than 40 percent of people aged 65 years or older experience one or more falls each year, often resulting in injuries [1,2]. Consequences of falls include a reduction in physical activity and functional decline which in turn can lead to a poorer quality of life and social isolation. All these factors increase the risk of dependency and institutionalisation [3-5].

Many older people do not seek medical attention after a fall [6] but a significant and increasing number require paramedic attendance as a consequence of a fall. In the period July 2008 – June 2009, the Ambulance Service of New South Wales (ASNSW), Australia attended to 42,331 fallers aged 65 years and older which constitutes approximately 5.1% of all emergency ambulance responses [7]. This proportion is similar to the 7.5% reported in an urban Emergency Medical Service system in the USA [8]. The demand for emergency services to assist older fallers is likely to increase with population ageing.

In most countries, the current practice for ambulance service paramedics is to convey older people who fall to the hospital Emergency Department (ED) unless the person refuses or declines transport. Fallers account for almost one fifth of ED presentations by older adults [1,9] and in the absence of physical injury, abnormal physiological parameters or change in usual functional status, it could be argued that routine conveyance by ambulance to the ED is not the most effective or efficient use of resources. Furthermore, ED personnel face significant time constraints and therefore focus on addressing the immediate consequences of a fall which leaves little scope for considering a person’s risk of future falls and the provision of appropriate prevention strategies.

Currently the ASNSW has a 25% non-conveyance rate for older fallers which is similar to that reported by ambulance services in other countries [34% in the United Kingdom (UK) [10], 40% in the United States of America [8]. A number of articles have described non-conveyance to be due to the treatment on scene being “sufficient” or the person requiring “lift assist only” [10-14]. What happens to these older fallers subsequent to the acute event is unclear, although a study from the UK highlighted substantial unplanned healthcare contact within two weeks, with 47% of these people calling the ambulance service again and 24% presenting to an Emergency Department [15]. An independent clinical review of these cases demonstrated that paramedics were almost certainly making correct decisions about conveyance at the time of assessment, but functional declines as a result of the fall frequently occurred 1–2 days later [15]. A recently completed study in New South Wales, Australia has also highlighted the high-risk nature of non-conveyed older fallers, demonstrating that within a 6 month follow-up period 58% of people fell again, 40% sustained a fall-related injury and 27% called an ambulance again due to a fall [16].

Rapid referral and timely access to alternate services to ED was identified as a gap in service provision in the UK based study and there is now evidence that an individualised multifactorial fall prevention program provided by community falls teams has significant benefits for non-conveyed older fallers [17]. This approach to intervention is consistent with the UK clinical fall guidelines [18] and included strength and balance training, assessment and modification of home hazards and medication review. The intervention group experienced significantly fewer falls (incidence rate ratio 0.45, 95% confidence interval (CI) 0.35 to 0.58) and the time to first subsequent fall was also significantly longer (median difference 145 days, HR 0.32, 95% CI 0.23 to 0.44). Furthermore, the intervention group made significantly fewer emergency calls for ambulance services within the follow-up period, compared to the control group participants who received standard emergency care (IRR 0.60, 95% CI 0.40 to 0.92).

Older fallers who require ambulance care in NSW appear to be comparable to their counterparts in the UK regarding level of fall risk, so it is likely that non-conveyed older fallers living in NSW also have the potential to benefit from timely access to fall risk assessment and intervention. The aim of this randomised controlled trial is to evaluate the effect of a rapid, timely and tailored intervention in older people who are not conveyed to a hospital ED following a fall.

The impact of the project will be evaluated over 12 months using a) rate of falls and fall-related injury, b) use of Ambulance resources, c) use of ED and in-patient services, d) incremental costs (or cost savings) of implementing this targeted approach, e) impact on the health care system (i.e. post acute care services (PACS) or Home Medication Review (HMR) services, etc.), f) uptake and adherence to recommendations and g) the benefits beyond fall prevention i.e. improved level of function and better quality of life. Importantly, rather than setting up a new service, our intervention will involve expanding existing services where possible, such as PACS which are capable of rapid responses to the urgent needs of community dwelling older people [19,20].

## Methods

### Design

A single blind randomised controlled trial will be conducted to evaluate the impact of rapid access to a comprehensive assessment and intervention program offered to non-conveyed older fallers. Subsequent falls and fall-related injuries, ambulance service use, ED and in-patient service use, incremental costs (or cost savings) of implementing this targeted approach, and impact on the health care system will be evaluated. In addition, we will investigate uptake and adherence to recommendations and benefits of the intervention beyond fall prevention.

### Participant selection and identification

Community dwelling people aged 65 years or older who receive ambulance care as a result of a fall and who are considered to be appropriate (or choose) to be left at home will be invited to participate in the trial. People living in Residential Aged Care Facilities will be excluded. People living at home with a known diagnosis of dementia and no carer will also be excluded as well as those who are unable to speak and understand sufficient English to participate fully in the trial.

All referred non-conveyed older fallers will be contacted via telephone to establish that there has been no acute deterioration in their health or level of function following the fall. For falls that occur between Monday and Thursday, calls will be made within 24 hours. For falls that occur between Friday and Sunday, calls will be made on the following Monday. Once it is established that there is no requirement for emergency care, the older person will be invited to participate in a fall prevention program. If verbal consent to participation is gained then an appointment for a home visit will be offered as soon as possible.

Ethics approval has been obtained from Sydney Local Health Network Ethics Review Committee (Royal Prince Alfred Hospital Zone- Protocol No X10-0352 & HREC/10/RPAH/616) and written consent will be obtained from all eligible participants.

### Ambulance station and paramedic participation

**Figure 1:**
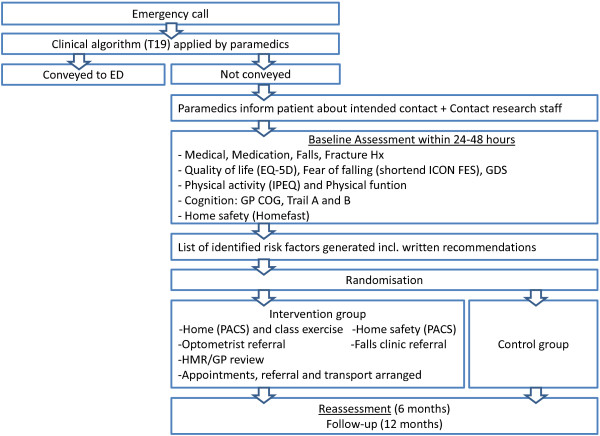
**Study outline; recruitment process and assessment.** Legend Figure 1: T19: Decision making protocol for paramedics when assessing falls in the elderly; ED: Emergency department; Hx: History; ICON FES: Iconographic falls efficacy scale; GDS: Geriatric depression scale; EQ-5D: Euro-Qol health questionnaire; IPEQ: Incidental and planned exercise questionnaire; GP COG: The General Practitioner assessment of Cognition; PACS: Post Acute Care Service; HMR: Home medication review; GP: General Practitioner.

The participating ambulance stations will be located in the eastern suburbs of Sydney, Australia and are largely within the Prince of Wales Hospital Post-Acute Care (POWH PACS) service catchment area (Randwick, Australia). All paramedics working at the participating stations will receive education with respect to the rationale for the study and will be trained in the process used to refer potentially eligible patients. Paramedic assessment will reflect existing standard practice within the ambulance service, which for this patient population is underpinned by an algorithmic clinical protocol (Protocol T19 – Falls in the elderly). This protocol provides decision making support for paramedics when determining whether patients require transport to hospital. If medically appropriate, as determined by the outcome of the algorithm, paramedics can recommend non-conveyance to older fallers. Patients are able to refuse or decline transport despite a recommendation for conveyance on the understanding they are able to give informed consent. When an older person is not conveyed to hospital, the paramedic will inform him/her that the Ambulance Service officer will contact them within 24 hours (72 hours if attended on a Friday). Should the person require care before then, they will be advised, as is current practice, to contact their local General Practitioner (GP) or call an ambulance if they feel this is required. The paramedics will then contact the study coordinator (via mobile phone with voicemail) to provide the contact details of the eligible older person they treated (Figure 1).

### Measurement and procedures

All eligible older fallers will be visited at home by a research physiotherapist. The program will be explained in detail to participants and written consent will be obtained prior to undertaking the baseline assessment. All participants will undergo two measurements: one on entry to the study (baseline assessment) and one after the 6-month intervention period. Table 1 highlights the measures to be undertaken. At the end of the baseline assessment, the researcher will generate a list of risk factors identified and written suggestions about how they might be addressed using a template with capacity for free text to individualise advice. All pre-test measures will be assessed prior to randomisation. Post-intervention period measures will be undertaken by personnel blinded to group allocation.

**Table 1:** List of measures to be collected at baseline assessment (BA) and at reassessment (RA)

	**BA**	**RA**	**O**
**Socio-demographics**			
Age, gender, education, occupation, place and type of residence and number of co-habitants.	✓	**✗**	**✗**
**General health and function**			
Detailed information regarding non-transported fall.	✓	**✗**	**✗**
Information regarding falls and fractures in previous 12 months.	✓	**✗**	**✗**
Ambulance service use and hospitalisation in last 12 months (in general, and due to falls).	✓	**✗**	**✗**
Disease history of previous 12 months (Multipurpose Australian Co-morbidity Scoring Scale (MACSS)).	✓	**✗**	**✗**
Medication use.	✓	✓	**S**
Assistive walking device (indoor and outdoor use), need for assistance when performing seven Instrumental Activities of Daily Living (IADL).	✓	✓	**S**
The Incidental and Planned Exercise Questionnaire (IPEQ) will provide estimates of the frequency and duration of planned and incidental exercise [21].	✓	✓	**S**
Self-reported fear of falling and balance ability on a 5-point Likert-scale.	✓	✓	**S**
**Quality of life**			
The EuroQol-5D is a widely used utility-based quality of life instrument for estimating Quality Adjusted Life Years (QALYs) for economic evaluations [22]. It provides a simple descriptive profile and a single index value for health related quality of life.	✓	✓	**S**
**Neuropsychological**			
Fear of falling will be assessed using the Iconographical Falls Efficacy Scale-Short version (ICON FES) [23].	✓	✓	**S**
The 15-item Geriatric Depression Scale (GDS) will assess symptoms of depression [24,25].	✓	✓	**S**
General Practitioner assessment of COGnition (GPCOG) will provide a global measure of cognition [26] The GPCOG is a reliable, valid and efficient tool to quickly screen for dementia, which has been shown not to be influenced by the cultural or linguistic background of the person being assessed.	✓	✓	**S**
Executive Function (working memory, set shifting and response inhibition) will be assessed using the Trail-Making Test A and B (TMT A/B ) [27,28].	✓	✓	**S**
**Physical**			
Objective measure of falls risk using the QuickScreen [29]. This is a multifactorial, reliable and externally validated falls risk assessment tool. It is able to predict future fall risk with an accuracy of 72%.	✓	✓	**S**
Timed up and Go [30] and the co-ordinated stability test [31].	✓	✓	**S**
**Home environment**			
The HOME FAST assessment score is a valid predictor of falls, with an increased risk of 1-2% for every additional point scored on the scale [32].	✓	✓	**S**
**Follow-up- 12 months**			
Falls (monthly diaries) [33].			**P**
Subsequent use of health services (differentiation between fall-related or other medical reason) collected from monthly diary and with further telephone call for clarification if required.			**S**
GP visit, Specialist medical practitioners visit, ambulance service use, ED presentation, hospital admission, physiotherapy, occupational therapy.			
Number of falls requiring ED attendance and/or hospitalisation collected from monthly diary and with further telephone call for clarification if required.			**S**
Use of NSW Ambulance Service, including time on scene (routinely collected Ambulance Service data).			**S**
Any ED presentation or hospitalisation including Diagnosis Related Group (DRG) and Length of Stay (LOS) data (measured using falls calendar data and corroborated through the NSW Admitted Patient Data Collection).			**S**
Use of any community health services – GP visits, Home medication review (HMR), Aged Care Assessment Team (ACAT) referrals, use of Post Acute Care Service (PACS) services etc. (measured using monthly falls calendar data).			**S**
Uptake and adherence to recommendations (based on initial and post-intervention assessments).	**✗**	✓	**S**

*BA* Baseline Assessment, *RA* Reassessment, *O* Outcome measure, *S* secondary, *P *Primary.

### Randomisation

Participants will be randomised after the baseline assessment. Randomisation will be achieved using computer-generated random numbers and randomly permuted block sizes of 4–8. The single centralised randomisation schedule will be undertaken by the NeuRA research studies coordinator with no involvement in the study and group allocation will be concealed from all study investigators and staff using consecutively numbered sealed opaque envelopes.

### Intervention group

Participants in the intervention group will receive fall prevention strategies based on their individual risk factors identified. The clinical researcher will pro-actively work with the older person to prioritise and facilitate implementation of the suggested intervention strategies.

#### ***Physical performance***

Strength and balance training will be recommended if the participant’s physical performance is thought to be a contributing factor as assessed using the measures outlined in Table 1. It is anticipated that the majority of the intervention group will be offered this intervention. If the participant lives within the catchment area of the Prince of Wales Hospital, the Otago Exercise Programme [34] will be delivered by the PACS service physiotherapist or nurse. The Post Acute Care Service is a State funded clinical program that aims to provide rapid assessment and support of people through a range of disciplines to prevent unnecessary hospital admission or facilitate timely hospital discharge. The Ambulance Service do not currently access this service. Participants will also be referred to local evidence-based exercise programs (not all delivered through health care) to ensure that they continue exercising on completion of the Otago Exercise Programme. Participants living outside the catchment area will be offered the Otago Exercise Programme delivered by a research physiotherapist.

#### ***Home safety***

Issues related to safety in the home environment will be referred to the PACS occupational therapist. Where participants are residing outside of the PACS catchment area, the clinical researcher will arrange for a community occupational therapist from the relevant local hospital to address the identified safety issues.

#### ***Visual impairment***

People with poor vision and no associated clinical diagnosis or recent visual assessment will be referred to their usual eye-care provider or a local optometrist for a visual assessment. Participants will also be offered the option of a home visit by a community optometrist, and the clinical researcher will arrange the referrals if required.

#### ***Home medication review***

Participants taking medications known to increase fall risk will be referred to their GP and encouraged to discuss the ongoing need for these medications. These medications include benzodiazepines, antidepressants, antipsychotics and opiate containing analgesic agents. A Home Medication Review will also be encouraged particularly where there is evidence of impaired cognition or reported difficulties taking medications. The GP will be contacted by the researcher to explain the nature of the project and a referral template will be faxed to the GP to minimise any additional work for GPs.

#### ***Falls, balance and bone health clinic or aged care clinic***

For participants with complex needs or multiple identified risk factors, a referral to the POW Falls, Balance and Bone Health Clinic or Aged Care Clinic will be organised. The GP will be contacted by the researcher to explain the nature of the project and a referral template will be faxed to the GP to aid with the referral process and minimise additional work for the GP.

### Control group

Participants randomised to the control group will receive a written copy of the risk factors identified during the baseline assessment but will not be provided with any additional support to implement the recommendations. They will be encouraged to discuss the results of the baseline assessment with their GP or other relevant health care provider.

### Outcome measures

The primary and secondary study outcome measures are listed in Table 1. All participants will be followed for a total of 12 months after the baseline assessment to record the number of falls and use of emergency health care services. The primary outcome measure will be rate of falls over the 12 month follow up period. A fall will be defined as “unintentionally coming to the ground, floor or lower level” [33] and will be recorded on monthly fall calendars which participants will receive after the baseline assessment. Both intervention and control participants will be asked to record the following details regarding falls: number of falls experienced during that month, location of the fall/s (indoor or outdoor fall) and injuries suffered. If calendars are not returned, telephone contact will be made to obtain the relevant information. This method for collection of fall information is recommended as best practice by the Prevention of Falls Network Europe (ProFaNE) [33].

### Statistical analysis

Analyses will be conducted using SPSS version 20.0 (SPSS Corp, Chicago, Ill, USA) and STATA 12 software (Stata Corp, College Station, Tex., USA). Descriptive statistics will be used to summarise demographic characteristics and baseline data. Proportion of fallers will be compared between groups using the Relative Risk statistic. Fall rates will be compared between groups using Incidence Rate Ratios (IRR) from negative binomial regression models [35]. Between-group comparisons of final EQ-5D (and other continuous measures) will be made using General Linear Models (ANCOVA) controlling for baseline performance and other confounding variables if required. Predictors of adoption and adherence will be analysed using multivariate modelling techniques. An intention-to-treat approach will be used for all between-group analyses.

### Economic analysis

Analysis will be undertaken in the manner used in previous fall prevention studies led by Campbell and Robertson [35,36]. The study will collect data on the cost to deliver the assessment and intervention package (including staff costs, training, capital costs and consumables), in-patient hospital admissions, ED presentations and other health and community service contact, fall rates and utility-based quality of life. Data linkage of all trial participants via CHeReL (Centre for Health Record Linkage) will be used to estimate the resource use associated with inpatient and ED visits. Incremental cost-effectiveness ratios will be calculated in terms of a) the incremental cost per fall prevented, b) the incremental cost per triple zero call avoided, c) the incremental cost per ED presentation avoided, and d) the incremental cost per hospital admission avoided and e) the incremental cost per QALY gained. Using the mean costs in the intervention and control arms, and the mean health outcomes in the control and intervention groups, the incremental cost per additional health outcome gained (outcomes a-e above) of the intervention group compared to control group will be calculated; results will be plotted on a cost-effectiveness plane. Bootstrapping will be used to estimate a distribution around costs and health outcomes and to calculate the confidence intervals around the incremental cost-effectiveness ratio. One way sensitivity analysis will be conducted around key variables, and a probabilistic sensitivity analysis will be conducted to estimate the joint uncertainty in all parameters; a cost-effectiveness acceptability curve (CEAC) will be plotted. A CEAC provides information about the probability that an intervention is cost-effective, given a decision maker’s willingness to pay for each additional health outcome.

### Sample size

The primary outcome measure will be the proportion of participants who fall during the twelve month follow-up period. Based on the fall rate from similar work in the field we estimate the fall rate in the control arm of this study will be 60%. We further estimate that the intervention will reduce the number of fallers by 33% in this period – a conservative estimate given the 55% reduction in fall rates reported in the UK over 1 year for a study with a similar intervention [37]. Consequently, accounting for dropouts (10%), a power of 90% and a 5% significance level, a total sample size of 234 is required. Based on our previous studies, a sample of 234 will also be adequate for determining clinically significant differences between the groups for our continuously scored measures [38], and for developing multivariate models pertaining to uptake and adherence [39].

## Discussion

A lack of evidence regarding the effectiveness or feasibility of alternate models of care following ambulance non-conveyance of older fallers, has resulted in a need to explore assessment and intervention programs to help reduce subsequent falls, related injuries and subsequent use of health care services. Previous studies conducted in the ED with patients presenting with a fall have demonstrated benefits of a multifactorial approach to intervention in terms of reducing falls [40] and the risk of functional impairment [41]. Recent evidence from the UK specifically addressing interventions offered to non-conveyed fallers also supports this multidisciplinary approach and demonstrated a significant reduction in falls and a positive effect on patient health outcomes as a result of the intervention [17].

This study is putting in place an approach to intervention that is being tested in a different health system in a different part of the world and where possible is using a service linking approach with existing services rather than setting up a new team. The approach is consistent with National Guidelines [18]. By linking existing services rather than setting up new services, this pragmatic trial aims to utilise the existing health care system infrastructure in an efficient and timely manner and has the potential benefit of being both generalizable and sustainable.

The feasibility of the project is contingent on a number of factors including paramedic referral of eligible participants, participants’ willingness to enrol into the study, GPs to provide necessary referrals and participant adherence to the intervention recommendations. A number of strategies will be employed to engage with the paramedics so as to optimise recruitment including regular station meetings, electronic communication and feedback on the types of intervention being offered to participants they have referred. Telephone communication will be used for GPs when seeking referrals to various parts of the healthcare system. Lastly, by staying in regular contact with all participants and providing timely and ongoing support we hope to achieve high adherence rates. Adherence to a home-based exercise program has been shown to be higher compared to group-based exercise programs among older people [42]. Adherence will also be maximised by using written descriptions [43], training diaries [44] and continued support through telephone contact from the exercise instructor [45], in accordance with the Otago Exercise Programme protocol.

The design of the study is such that, if effective, it would be relatively easy to embed into normal practice as the approach used is focusing on processes to link existing services rather than creating new services.

## Conclusion

The aim of this randomised controlled trial is to evaluate the effect of a rapid, timely and tailored intervention in older people who are not conveyed to ED following a fall. The impact will be assessed based on future falls, fall-related injuries and subsequent use of emergency services. Additionally this trial will determine the effectiveness of pro-active interventions when addressing individual fall risk factors compared to standardized recommendations. We aim to better understand how to optimise referral pathways and clinical care for older people who experience a fall and are not conveyed to hospital.

## Abbreviations

ACAT: Aged care assessment team; ASNSW: Ambulance Service of New South Wales; CHeReL: Centre for health record linkage; DRG: Diagnosis related group; ED: Emergency department; GP: General practitioner; GP Cog: The general practitioner assessment of cognition; HMR: Home medication review; IADL: Instrumental activities of daily living; ICON: FES iconographic falls efficacy scale; IPEQ: Incidental and planned exercise questionnaire; LOS: Length of stay; MACSS: Multipurpose Australian Co-morbidity scoring scale; NSW: New South Wales, Australia; PACS: Post acute care service; POWH: Prince of Wales hospital, Randwick, Australia; QALY: Quality adjusted life years; TMT A/B: Trail making test A and B; UK: United Kingdom.

## Competing interests

The authors declare that they have no competing interests.

## Authors’ contributions

AM and JC drafting of the manuscript. All authors contributed to study design and objectives and read and approved the final manuscript.

## Pre-publication history

The pre-publication history for this paper can be accessed here:

http://www.biomedcentral.com/1472-6963/13/360/prepub
